# Cobenfy (Xanomeline-Trospium Chloride): A New Frontier in Schizophrenia Management

**DOI:** 10.7759/cureus.71131

**Published:** 2024-10-09

**Authors:** Abdul Haseeb Hasan, Muhammad Ali Abid

**Affiliations:** 1 Medicine, Mayo Hospital, Lahore, PAK

**Keywords:** antipsychotics, cobenfy, emergent, schizophrenia, trospium chloride, xanomeline, xanomeline-trospium

## Abstract

Schizophrenia is a complex mental disorder characterized by psychotic symptoms that significantly impair social and occupational functioning. The traditional treatment approach has relied on antipsychotics that primarily target dopamine receptors. However, these medications often come with notable limitations and side effects. Recently, Cobenfy, a novel antipsychotic combining xanomeline and trospium chloride, received the United States (US) Food and Drug Administration (FDA) approval, marking a significant advancement in schizophrenia treatment. This first-in-class medication operates through a unique mechanism, activating cholinergic receptors to mitigate psychotic symptoms while minimizing the common adverse effects associated with dopamine-blocking agents. Clinical trials, particularly the EMERGENT-2 and EMERGENT-3 studies, demonstrated that Cobenfy substantially improved both positive and negative symptoms of schizophrenia, achieving greater reductions in the Positive and Negative Syndrome Scale (PANSS) total score compared to placebo. Moreover, Cobenfy exhibited a favorable safety profile, with fewer incidences of weight gain and extrapyramidal symptoms. However, it is associated with side effects such as nausea, dyspepsia, and constipation. It also carries risks for specific patient populations, such as patients with hepatic or renal dysfunction. Overall, Cobenfy offers a promising alternative for individuals with schizophrenia, particularly those who are treatment-resistant or experience intolerable side effects from conventional therapies. Continued evaluation of its long-term efficacy and safety is essential, along with monitoring for potential adverse effects in vulnerable populations.

## Editorial

Schizophrenia is a severe mental disorder characterized by psychotic symptoms such as hallucinations, delusions, and cognitive dysfunction [1]. It frequently leads to a decline in social and occupational functioning, with many individuals experiencing persistent disability [1]. Despite common misconceptions, the disorder is slightly more prevalent in men, and outcomes vary significantly [1]. Schizophrenia is categorized into positive symptoms (e.g., hallucinations), negative symptoms (e.g., social withdrawal), and disorganization syndromes (e.g., incoherent speech) [1]. Over the years, significant brain structure abnormalities, including lateral ventricular enlargement and reduced brain volume, have been associated with the disorder, further supporting its complexity [1]. Current evidence suggests neurochemical disturbances, particularly in dopamine and glutamatergic systems, are central to the pathophysiology of schizophrenia [1].

Moreover, the genetic component of schizophrenia is now understood to be largely polygenic, with birth and early life factors playing a role in its development [1]. Although treatment has traditionally relied on antipsychotic drugs that block dopamine receptors, a significant challenge remains in managing schizophrenia due to the high rate of treatment resistance, which affects up to 34% of patients [2]. This treatment resistance further compounds the societal burden of schizophrenia, a condition that continues to have a profound global impact. Between 1990 and 2019, disability-adjusted life years (DALYs) associated with schizophrenia increased by 65%, reflecting the substantial toll this disorder takes on individuals and healthcare systems worldwide [2]. Moreover, over the past two decades, both the incidence and prevalence of schizophrenia have risen dramatically by 37 and 65%, respectively [2]. These statistics highlight the urgent need for more effective therapeutic strategies to address the limitations of current treatments and improve patient outcomes [1,2].

A novel approach to schizophrenia treatment has emerged with the development of the drug Cobenfy, recently approved by the United States (US) Food and Drug Administration (FDA) [3]. This first-in-class antipsychotic offers a unique mechanism of action by targeting cholinergic receptors rather than the traditional dopamine receptors. Cobenfy combines xanomeline, an oral muscarinic cholinergic receptor agonist, with trospium chloride, a peripheral muscarinic receptor antagonist [2,3]. This innovative combination addresses the growing need for new therapeutic options that avoid the limitations of existing antipsychotics. Xanomeline, previously investigated for Alzheimer's disease, has demonstrated effectiveness in modulating muscarinic receptors in the brain, particularly the M1 and M4 subtypes, which play a role in cognitive and psychotic symptom regulation. Trospium, initially developed to treat overactive bladder, serves as a peripheral antagonist to reduce the side effects of xanomeline without compromising its central action [2].

The FDA approval of Cobenfy was largely based on the results of the EMERGENT clinical trials, which assessed the efficacy and safety of xanomeline-trospium in patients with schizophrenia [3]. These trials, including EMERGENT-2 and EMERGENT-3, enrolled participants with schizophrenia who were experiencing acute psychosis and compared the effects of xanomeline-trospium to placebo over five weeks [3-5]. The primary outcome measure in these studies was the change in the Positive and Negative Syndrome Scale (PANSS) total score from baseline to week 5 [3-5]. In the pivotal trials, patients treated with Cobenfy showed substantial improvement in both positive and negative symptoms of schizophrenia, with an average reduction of 9.6 and 8.4 points on the PANSS score in the respective trials [3-5]. Secondary endpoints, such as overall clinical severity, also showed marked improvements. Importantly, the drug was associated with better tolerability compared to existing antipsychotics with fewer side effects, such as weight gain or extrapyramidal symptoms, commonly seen with dopamine-blocking therapies [3-5].

However, Cobenfy is not without adverse effects [3]. The most frequently reported side effects included nausea, dyspepsia, constipation, vomiting, and hypertension [3-5]. More concerning, though rare, were reports of urinary retention, increased heart rate, and reduced gastrointestinal movement [3,5]. The drug also carries a risk of liver damage, and patients with moderate to severe renal or hepatic impairment are advised against its use [3]. Despite these potential risks, Cobenfy presents a significant advantage over traditional treatments by not contributing to weight gain or motor side effects, two major concerns with dopamine-targeting antipsychotics [3-5]. Furthermore, its unique action on the cholinergic system offers a promising alternative for patients who do not respond to dopamine receptor antagonists.

Given these promising results, Cobenfy represents a breakthrough in the management of schizophrenia. However, ongoing monitoring of its long-term safety and effectiveness is essential. Recommendations for future use include regular screening for hepatic and renal function in patients, particularly those with pre-existing conditions. Clinicians should also monitor for signs of cholinergic overload, especially in individuals with conditions such as glaucoma or gastrointestinal disorders. Overall, the approval of Cobenfy marks an important step in advancing treatment options for schizophrenia, offering hope to patients who have struggled with treatment resistance and adverse effects from conventional therapies.

## References

[1] Jauhar S, Johnstone M, McKenna PJ (2022). Schizophrenia. The Lancet.

[2] Vasiliu O, Budeanu B, Cătănescu MȘ (2024). The new horizon of antipsychotics beyond the classic dopaminergic hypothesis—the case of the xanomeline-trospium combination: a systematic review. Pharmaceuticals (Basel).

[3] (2024). FDA OKs first-in-class antipsychotic for schizophrenia. https://www.medscape.com/viewarticle/fda-oks-first-class-antipsychotic-schizophrenia-2024a1000hno.

[4] Kaul I, Sawchak S, Correll CU (2024). Efficacy and safety of the muscarinic receptor agonist KarXT (xanomeline-trospium) in schizophrenia (EMERGENT-2) in the USA: results from a randomised, double-blind, placebo-controlled, flexible-dose phase 3 trial. Lancet Lond Engl.

[5] Kaul I, Sawchak S, Walling DP (2024). Efficacy and safety of xanomeline-trospium chloride in schizophrenia: a randomized clinical trial. JAMA Psychiatry.

